# Supplementary material for: Altered microRNA Transcriptome in Cultured Human Liver Cells upon Infection with Ebola Virus
Source: Int J Mol Sci. 2021 Apr 6;22(7):3792. doi: 10.3390/ijms22073792 (PMC8038836; doi:10.3390/ijms22073792)
Supplement: Supplementary file 1 [file ijms-22-03792-s001.zip › Supplementary_File/C_ GO_Analysis_Results/16-30nt_go_Mayinga-24h-Huh7_vs_Control-24h-Huh7_down.mature_mirna_targets/BP_Pvalue_tree.pdf]

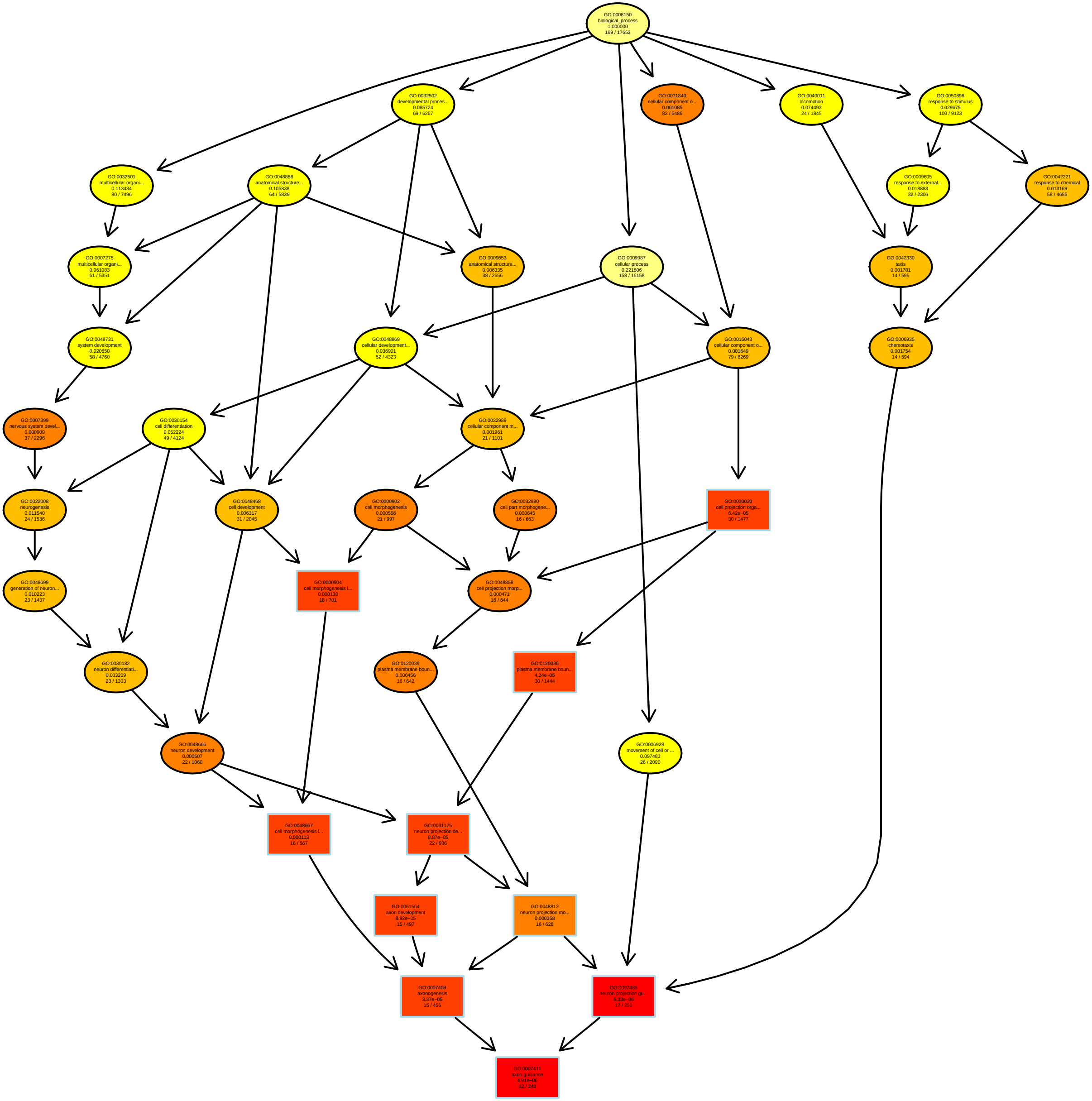
GO:0008150 biological_process 1.000000 169 / 17653
GO:0032502 developmental proces... 0.085724 69 / 6267
GO:0071840 cellular component o... 0.001085 82 / 6486
GO:0040011 locomotion 0.074493 24 / 1845
GO:0050896 response to stimulus 0.029675 100 / 9123
GO:0032501 multicellular organi... 0.113434 80 / 7496
GO:0048856 anatomical structure... 0.105838 64 / 5836
GO:0009605 response to external... 0.018883 32 / 2306
GO:0042221 response to chemical 0.013169 58 / 4655
GO:0007275 multicellular organi... 0.061083 61 / 5351
GO:0009653 anatomical structure... 0.006335 38 / 2656
GO:0009987 cellular process 0.221806 158 / 16158
GO:0042330 taxis 0.001781 14 / 595
GO:0048731 system development 0.020650 58 / 4760
GO:0048869 cellular development... 0.036901 52 / 4323
GO:0016043 cellular component o... 0.001649 79 / 6269
GO:0006935 chemotaxis 0.001754 14 / 594
GO:0007399 nervous system devel... 0.000909 37 / 2296
GO:0030154 cell differentiation 0.052224 49 / 4124
GO:0032989 cellular component m... 0.001961 21 / 1101
GO:0022008 neurogenesis 0.011540 24 / 1536
GO:0048468 cell development 0.006317 31 / 2045
GO:0000902 cell morphogenesis 0.000566 21 / 997
GO:0032990 cell part morphogene... 0.000645 16 / 663
GO:0030030 cell projection orga... 6.42e−05 30 / 1477
GO:0048699 generation of neuron... 0.010223 23 / 1437
GO:0000904 cell morphogenesis i... 0.000138 18 / 701
GO:0048858 cell projection morp... 0.000471 16 / 644
GO:0030182 neuron differentiati... 0.003209 23 / 1303
GO:0120039 plasma membrane boun... 0.000456 16 / 642
GO:0120036 plasma membrane boun... 4.24e−05 30 / 1444
GO:0048666 neuron development 0.000507 22 / 1060
GO:0006928 movement of cell or ... 0.097483 26 / 2090
GO:0048667 cell morphogenesis i... 0.000113 16 / 567
GO:0031175 neuron projection de... 8.87e−05 22 / 936
GO:0061564 axon development 8.92e−05 15 / 497
GO:0048812 neuron projection mo... 0.000358 16 / 628
GO:0007409 axonogenesis 3.37e−05 15 / 456
GO:0097485 neuron projection gu... 5.33e−06 12 / 250
GO:0007411 axon guidance 4.91e−06 12 / 248
